# Microtubule organization is determined by the shape of epithelial cells

**DOI:** 10.1038/ncomms13172

**Published:** 2016-10-25

**Authors:** Juan Manuel Gomez, Lyubov Chumakova, Natalia A. Bulgakova, Nicholas H. Brown

**Affiliations:** 1 Department of Physiology, Development and Neuroscience, and the Gurdon Institute, The University of Cambridge, Cambridge CB2 3DY, UK; 2 School of Mathematics and Maxwell Institute for Mathematical Sciences, The University of Edinburgh, Edinburgh EH9 3FD, UK

## Abstract

Interphase microtubule organization is critical for cell function and tissue architecture. In general, physical mechanisms are sufficient to drive microtubule organization in single cells, whereas cells within tissues are thought to utilize signalling mechanisms. By improving the imaging and quantitation of microtubule alignment within developing *Drosophila* embryos, here we demonstrate that microtubule alignment underneath the apical surface of epithelial cells follows cell shape. During development, epidermal cell elongation and microtubule alignment occur simultaneously, but by perturbing cell shape, we discover that microtubule organization responds to cell shape, rather than the converse. A simple set of microtubule behaviour rules is sufficient for a computer model to mimic the observed responses to changes in cell surface geometry. Moreover, we show that microtubules colliding with cell boundaries zip-up or depolymerize in an angle-dependent manner, as predicted by the model. Finally, we show microtubule alignment responds to cell shape in diverse epithelia.

## Abstract (web summary)

Interphase microtubule organization creates a structural scaffold for cargo delivery determining cell polarity and cell shape. Here the authors show that in *Drosophila* epithelia the orientation of microtubules responds to cell shape, rather than the converse, and develop a simple model to mimic this behaviour.

## Graphical abstract

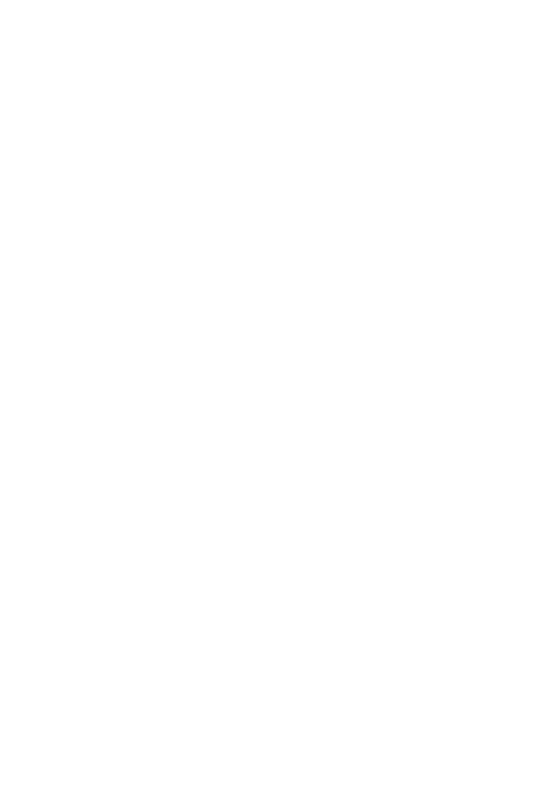

Interphase microtubule (MT) organization is diverse across tissues and cell types, ranging from radial patterns to parallel arrays (referred as ‘aligned MTs'). Aligned MTs are a hallmark of specialized cell types with non-centrosomal MTs, such as neuronal and epithelial cells^1^,^2^. The MT alignment creates a structural scaffold for vectorial transport of different cargos, and therefore plays important functions in cell polarity, cell shape and cell migration^3^. Recently, we discovered that cell–cell contact stability and cell movements within the epidermis of *Drosophila* embryos are regulated by an asymmetric localization of E-cadherin, which requires an aligned array of MTs (ref. 4). Therefore, we sought to discover how MTs become aligned in this epithelial cell layer.

In single cells, whether yeast or mammalian cells in culture, MT organization can be explained by a physical mechanism where MTs follow changes in cell geometry^5^,^6^,^7^. Cell geometry acts on MT organization largely by altering the dynamics of the MTs colliding with the cell boundary. At the cell boundary, MTs either depolymerize (undergo catastrophe), become more dynamic (undergo cycles of growth/shrinkage) or become tethered to the cell boundary^8^,^9^. In yeast, the instability of MTs has been linked to the compressive forces generated by growing MTs against the cell boundary, which in turn reduce MT growth speed and enhance their catastrophe frequencies^10^. In contrast, MTs in cells within multicellular tissues appear to be organized by signalling mechanisms. Work in *Drosophila* suggests that the planar cell polarity (PCP) pathway components Fat and Dachsous regulate MT organization in animal epithelial tissues^11^,^12^,^13^, suggesting that MT behaviour is differentially regulated around the cell periphery. In plants, the orientation of interphase MTs has been shown to be actively regulated by a variety of mechanisms, such as inducing asymmetric MT nucleation, altering the direction of initial MT growth, stabilizing MT plus-ends at specific cell boundaries and Katanin-mediated severing of MTs^14^,^15^,^16^,^17^.

Here, we describe improvements in our ability to quantify MT alignment, by using 3D Structured Illumination Microscopy (3D-SIM) to generate high-resolution images of the MTs, and combining this with automated image analysis to quantify MT organization relative to cell shape. This analysis showed that even within a multicellular tissue MT alignment correlated well with cell shape, suggesting that a PCP mechanism may not be necessary. Experimental perturbation of cell shape or MTs showed MTs responding to cell shape changes, whereas cell shape was remarkably impervious to loss of MTs. Using computer modelling we explored the constraints on MT behaviour that are required to mimic the observed correlation between the geometry of the apical surface and MT organization. By analysing EB1-green fluorescent protein (GFP) trajectories and collisions with the cell membrane, we were able to confirm the angle-dependence of MT–cell boundary collisions (zipping-up versus catastrophes), a mechanism predicted to contribute to MT alignment by the computer model. Altogether, this demonstrates that the organization of MT arrays within epithelial layers of *Drosophila* can be largely explained by the response of growing MTs to the geometric constraints of the cell.

## Results

### MT reorganization and cell elongation occur concomitantly

To investigate the mechanism of MT organization, we combined 3D-SIM super-resolution imaging with automated image analysis of cell shape and MTs within each cell (see the ‘Methods section', Supplementary Fig. 1a–d). To analyse cell shape, we fit the cell to an ellipse to measure the degree of cell elongation (eccentricity, Supplementary Fig. 1c,g), determine the major and minor axes of the ellipse (referred as cell's long and short axes) and measure their length and the ratio between them (Supplementary Fig. 1c). To analyse MTs, we quantified their organization using two parameters: the deviation of the average direction of the MTs from the long axis of the cell (‘MT Deviation', MTDEV; Supplementary Fig. 1d), which will become smaller as the MTs become oriented parallel to the long axis, and the degree of MT alignment with each other by measuring the degree of deviation from the mean direction of α-Tubulin signal (‘MT alignment', MTSD for MT s.d., Supplementary Fig. 1d), which also reduces as MTs become aligned with each other (Supplementary Fig. 1g).

**Figure 1:**
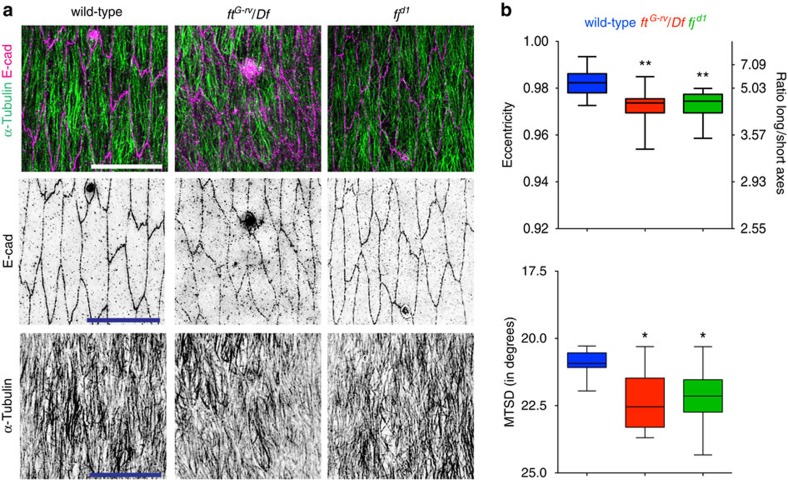
Loss of function of PCP pathway components Fat and Four-Jointed results in weak defects of cell shape and MT organization. (**a**) Dorso-lateral regions of stage 15 embryonic epidermis, with apical cell outlines revealed with an E-cadherin (E-cad) antibody (magenta) and MTs with an α-Tubulin antibody (green): wild-type (left), *fat* mutant over *fat* deficiency *(ft*^*G-rv*^/*Df*; centre) and *four-jointed* mutant (*fj*^*d1*^; right). Scale bars, 10 μm (**b**) Quantification of cell eccentricity and cell's long/short axes ratio (top) and MT alignment (MTSD; bottom). Cells from 12 embryos from 3 (wild type, *ft*^*G-rv*^/*Df*) and 2 (*fj*^*d1*^) independent experiments were analysed. Statistical analysis of data combined with data from Supplementary Fig. 2a,b was performed with one-way ANOVA and Fisher's LSD *post hoc* analysis. *P<0.05, **P<0.01.

**Figure 2:**
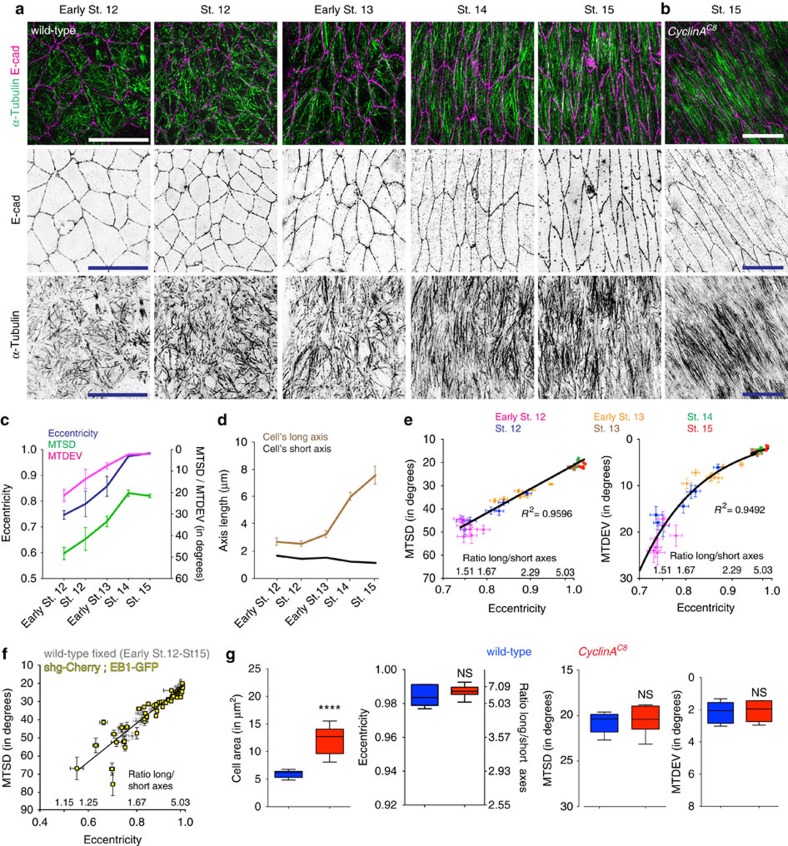
Changes in epidermal cell shape and MT organization correlate during embryogenesis. (**a**,**b**) Representative images of dorsal–lateral epidermis, visualized as described in Fig. 1, from wild-type embryos at the developmental stages and a *CyclinA*^*C8*^ mutant embryo, with the latter shown at lower magnification (**b**). Scale bars, 10 μm. (**c**,**d**) Quantification of cell eccentricity (**c**, blue), MT alignment (MTSD; **c**, green), alignment of MTs with the long axis of the cell (MTDEV; **c**, magenta), cell's long (**d**, brown) and short (**d**, black) axis length in wild-type embryos at the stages (St) shown. Cells from 7 embryos per developmental stage from 1 (Early St 12, St 13–15) and 2 (St 12 and Early St 13) independent experiments were analysed. Error bars show mean±s.d. (**e**) Correlation between MTSD (left) or MTDEV (right) with cell elongation (eccentricity/aspect ratio). Each dot represents cells from a single embryo (mean±s.e.m.). (**f**) Correlation between MTSD and elongation is the same in fixed embryos, in which alignment of MTs visualized by α-Tubulin staining was analysed (from **e**, grey), and live embryos expressing EB1-GFP (yellow), in which alignment of EB1 comet trajectories was analysed. Each dot in yellow represents a cell with mean±s.e.m. of MTSD and eccentricity from 10 consecutive time intervals. 58 cells from 8 embryos between St 12 and St 15 from three independent experiments were analysed. (**g**) Quantification of cell area, cell eccentricity and aspect ratio of long/short axes, MTSD and MTDEV in cells of wild-type (blue) and *CyclinA*^*C8*^ (red) stage 15 embryos. Cells from seven embryos per genotype from three independent experiments were analysed. Statistical analyses were performed with unpaired *t*-test. ****P<0.0001. NS, not significantly different.

To validate the Matlab script measurements, we compared MTSD values produced using three methods: cell-by-cell analysis using our Matlab script, and analysis of the entire field of cells using both the Matlab script and the Fiji Directionality tool used previously^18^ (Fourier components). Quantifications were done on cells in the dorsal–lateral epidermis of stage 15 wild-type *Drosophila* embryos, where arrays of sub-apical MTs are aligned and oriented parallel to the cell's long axis (Fig. 1a). All three methods produced values that were not significantly different (*P*=0.065, one-way analysis of variance (ANOVA), Supplementary Fig. 1e), and thus these independent methods are consistent. Additionally, it demonstrates that the shape of the masks used to extract each cell's α-Tubulin signal has no influence on the outcome, as both elongated cell masks and square field masks gave similar MTSD values. To further test our method, we quantified MT organization in stage 12 embryos, where MTs visually appear to be unaligned (Fig. 2a). In this case, analysis of the entire field with either the Matlab script or the Directionality tool produced the same MTSD values (*P*=0.459, one-way ANOVA, Supplementary Fig. 1e). However, when we compared cell-by-cell and entire field analyses with the Matlab script, the cell-by-cell analysis resulted in smaller and less spread MTSD values (*P*=0.028, one-way ANOVA and Fisher's LSD *post hoc* analysis, Supplementary Fig. 1e). This difference is likely to arise from the fact that the long axes of the cells are less aligned at stage 12 (Fig. 2a, Supplementary Fig. 1f), resulting in a misalignment of α-Tubulin signal. Therefore, we conclude that our method quantifies MT organization similarly to previously published approaches, but with an additional increase in accuracy from the cell-by-cell approach. As a further test, we generated images containing simulated MTs and confirmed that the calculated MTSD matched that measured by our script (Supplementary Fig. 1h).

Previous work has suggested that MT alignment in the ventral epidermis of *Drosophila* embryos requires the PCP pathway component Fat, an atypical cadherin^12^. We sought to confirm that Fat also has a role in MT alignment in the dorsal–lateral epidermis using stage 15 embryos, but were surprised to find only a modest reduction of MT alignment in the absence of Fat (7% MTSD increase, *P*=0.002, one-way ANOVA and Fisher's LSD *post hoc* analysis, Fig. 1a,b). We also noted that the dorsal–lateral epidermal cells were less elongated than wild-type (19% reduction in long/short axes, 1% reduction in eccentricity, *P*=0.0001, one-way ANOVA and Fisher's LSD *post hoc* analysis, Fig. 1a,b), which can also be seen in the ventral epidermis^12^. Note that the relationship between cell eccentricity and long/short axes ratio (aspect ratio) is non-linear, with large changes in the aspect ratio of the most elongated cells corresponding to small changes the eccentricity values as they approach 1. To test further the requirement of the PCP pathway for MT alignment, we tested PCP components that were either previously linked to MT organization in other epithelia or asymmetrically localized in the *Drosophila* embryonic epidermis^11^,^19^ (Fig. 1a,b, Supplementary Fig. 2a,b). Of these, only the loss of the Golgi kinase Four-jointed (*fj*^*d1*^) resulted in reduction of MT alignment, but also affected cell elongation (6% MTSD increase, *P*=0.005 and 19% aspect ratio reduction/1% eccentricity reduction, *P*=0.0002 respectively, one-way ANOVA and Fisher's LSD *post hoc* analysis, Fig. 1a,b), to a similar extent as the loss of Fat (*P*=0.743 and *P*=0.803 respectively, one-way ANOVA and Fisher's LSD *post hoc* analysis). Thus, loss of Fat or Four-Jointed had modest effects on MT alignment, but also on cell shape, suggesting that the PCP regulation of MTs could alter cell shape, or regulation of cell shape could alter MTs.

To explore the linkage between cell shape and MT organization, we first measured the changes in cell elongation and MT organization that occur during development. Epidermal cells start to elongate during stage 12, first by shortening their short axis and then from stage 13 onwards, largely by lengthening their long axis (Fig. 2a,c,d). Simultaneously, sub-apical MTs progressively reorganized from a random to an aligned pattern that was oriented parallel to the cell's long axis (Fig. 2a, Supplementary Movie 1). This change was quantified as a reduction of both MTSD and MTDEV (Fig. 2c). Next, we analysed if there was a clear association between the changes in MT organization (that is, MTSD and MTDEV) and cell elongation (eccentricity) by testing their correlation during development. The MTSD and cell eccentricity had a linear correlation during development (Fig. 2e), while the MTDEV showed an exponential correlation with cell eccentricity (Fig. 2e). We also tested if the same correlation between MTSD and eccentricity is observed in live epidermal cells by marking MT plus-ends with EB1-GFP^4^, and using the resulting trajectories of MT plus-end growth to quantify MT alignment (Supplementary Movie 1). We found that both eccentricity and MTSD values from live epidermal cells of stage 12, 13 and 15 embryos were similar to the values in fixed embryos and thus, displayed the same linear correlation (*P*=0.07, Prism GraphPad shared linear fit of correlation comparison, Fig. 2f). Thus, the strong correlations between MT organization parameters and the elongation of epidermal cells suggested that cell eccentricity is a predictor of sub-apical MT organization.

To test whether it is the degree of cell elongation or the specific cell dimensions that predicts MT organization, we used a null mutant in the gene encoding the cell cycle factor Cyclin A (*CycA*^*C8*^)^20^, in which cell size is increased due to a block in the last mitosis of embryonic development. Given that epidermal cells can still undergo cell division during stage 12 (Fig. 2a), we analysed stage 13 and 15 embryos and found that their cell area was doubled (*P*<0.0001 for both, unpaired *t*-test, Supplementary Fig. 2c,d and Fig. 2b,h) but cells elongated to the same degree (*P*=0.5 and *P*=0.33, respectively, unpaired *t*-test, Supplementary Fig. 2c,d and Fig. 2b,h). The MTs in these cells had the same MTSD (*P*=0.57 and *P*=0.74 respectively, unpaired *t*-test, Supplementary Fig. 2c,d and Fig. 2b,h) and MTDEV (*P*=0.25 and *P*=0.93, respectively, unpaired *t*-test, Supplementary Fig. 2c,d and Fig. 2b,h) as wild-type. Moreover, *CycA*^*C8*^ embryos retain the wild-type correlations between both MTSD and MTDEV, and eccentricity (*P*=0.06, Prism GraphPad shared linear fit of correlation comparison and *P*=0.22, Wilcoxon test, Supplementary Fig. 2e). Therefore, cell eccentricity, rather than specific cell dimensions, predicts sub-apical MT organization.

### Changing cell shape alters sub-apical MT organization

**Figure 3:**
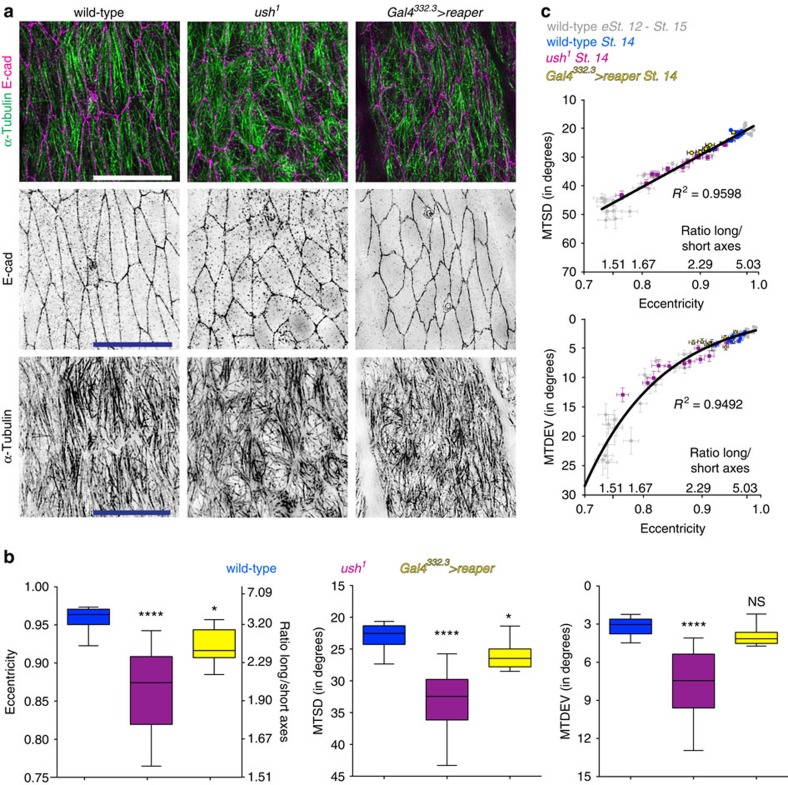
Changing cell shape alters sub-apical MT organization. (**a**) Representative images of dorsal–lateral epidermis, visualized as described in Fig. 1, of stage 14 wild type (left) and embryos with germband retraction blocked by the *ush*^*1*^ mutant (centre) embryos, or inducing cell death in the amnioserosa using *Gal4*^*332.3*^*>reaper* (right). Scale bars, 10 μm. (**b**) Quantification of cell eccentricity and cell's long/short axes ratio (left), MTSD (centre) and MTDEV (right) shows germband retraction is required for cell elongation and MT organization. Cells from 12 embryos (wild-type, *ush*^*1*^ mutant) and 10 embryos (*Gal4*^*332.3*^*>reaper*) from two independent experiments were analysed. Statistical analysis was performed with one-way ANOVA and Fisher's LSD *post hoc* analysis. *P<0.05, ****P<0.0001, NS, not significantly different. (**c**) Correlation between either MTSD (top) or MTDEV (bottom), and cell eccentricity is retained when cell elongation is blocked. Each dot represents the cells from a single embryo (mean±s.e.m.).

The correlation between cell eccentricity and MT organization could be causative, with one directing the other or each of them mutually affecting each other, or these two processes could be independent. Thus, we sought to test whether altering cell shape affects MT organization. The largest change in cell eccentricity and MT organization (Fig. 2a,c) occurred during the process of germband retraction, which involves a dramatic change in embryonic shape^21^. Therefore, to perturb the normal cell shape changes, we used a mutant that is unable to undergo germband retraction (*u-shaped*^*1*^ *(ush*^*1*^)), due to the lack of the U-shaped transcription factor essential for maintenance of the amnioserosa, an extraembryonic tissue required for germband retraction^22^,^23^. Stage 14 *ush*^*1*^ embryos had reduced cell elongation (43% reduction in aspect ratio/10% reduction in eccentricity), and increased MTSD (45%) and MTDEV (141%), compared with wild-type embryos of the same age (*P*<0.0001 for all three, one-way ANOVA and Fisher's LSD *post hoc* analysis, Fig. 3a,b). Furthermore, the correlation between eccentricity and MTSD or MTDEV in the cells of *ush*^*1*^ mutant embryos was the same as cells in wild-type embryos at earlier stages (*P*=0.51 for both, Prism GraphPad shared linear fit of correlation comparison -MTSD- and paired *t*-test-MTDEV, Fig. 3c).

As we could not rule out the possibility that U-shaped in some way directly alters MTs in the cells we are examining we utilized an independent way of impairing cell shape. We used a Gal4 driver just expressed in the amnioserosa cells^24^ (*Gal4*^*332.3*^), to induce their apoptosis by expression of the pro-apoptotic gene reaper^25^. The defects were less severe than in *ush*^*1*^ embryos, consistent with the weaker block of germband retraction (Fig. 3a, Supplementary Fig. 3a). Cell elongation and MTSD were impaired in *Gal4*^*332.3*^>*rpr* embryos (reduction of aspect ratio by 26% and eccentricity by 4%, *P*=0.02 and 14% MTSD increase, *P*=0.04 respectively, one-way ANOVA and Fisher's LSD *post hoc* analysis, Fig. 3a,b), while MTDEV was not significantly different from wild-type (*P*=0.3, one-way ANOVA and Fisher's LSD *post hoc* analysis, Fig. 3a,b), probably because cell elongation was not impaired to an extent where the alignment of MTs with the cell's long axis would fail. *Gal4*^*332.3*^>*rpr* embryos also maintained the same correlation between cell eccentricity and MTSD with both wild type and *ush*^*1*^ embryos (*P*=0.37, Prism GraphPad shared linear fit of correlation comparison, Fig. 3c). To examine whether *ush*^*1*^ or *Gal4*^*332.3*^>*rpr* had any direct effects on MTs, we analysed MT organization in early stage 12 *ush*^*1*^ and *Gal4*^*332.3*^>*rpr* embryos (Supplementary Fig. 3b), before any perturbation of cell shape. There was no significant change to MT organization (*P*=0.26 and *P*=0.8 for MTSD and MTDEV respectively, one-way ANOVA, Supplementary Fig. 3c), and as expected, cell eccentricity was not yet altered (*P*=0.22, one-way ANOVA). These results are consistent with the hypothesis that the physical constraints of the cell geometry could be sufficient to specify MT organization in these cells.

**Figure 4:**
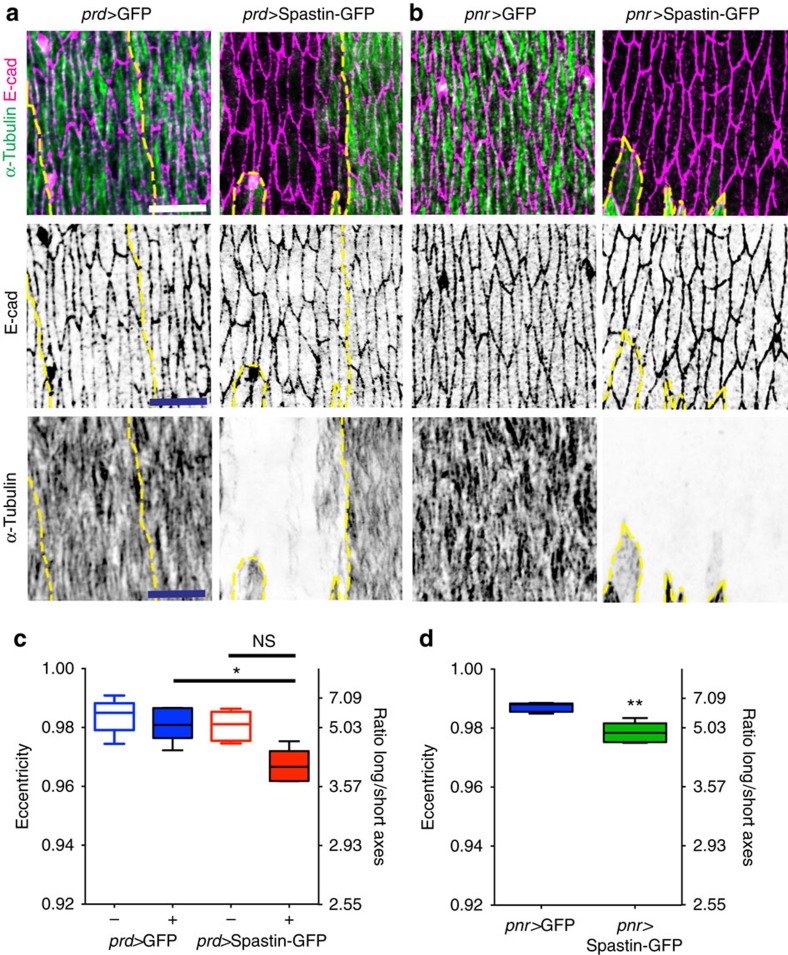
Eliminating MTs has minor effects on epidermal cell shape. (**a**,**b**) Representative images of dorsal–lateral epidermis, visualized as described in Fig. 1, of control embryos expressing GFP (left) or Spastin-GFP (right) with *paired*::*Gal4* (*prd>*, **a**) and *pannier*::*Gal4* (*pnr>*, **b**). Region expressing either GFP or Spastin-GFP is outlined with a yellow dotted line. Scale bars, 10 μm (**c**,**d**) Quantification of cell eccentricity and cell's long/short axes ratios in embryos expressing GFP (**c**, left and **d**, blue) and Spastin-GFP (**c**, right and **d**, green) shows that substantial cell elongation occurs in the absence of MTs. Cells from five embryos per genotype from 3 (**c**) and 2 (**d**) independent experiments were analysed. Statistical analysis between expressing (+) and non-expressing (−) cells within the same embryo in **c** was performed with Wilcoxon test and between cells from different embryos in **c** and **d** with Mann–Whitney test. *P<0.05 **P<0.01, NS, not significantly different.

We next assessed the converse, that is whether cell shape is dependent on MT organization. MTs were destroyed in epidermal cells, from early stage 12 onwards, by expressing the MT severing protein Spastin^26^ using the *paired::Gal4* driver, which is expressed in stripes along the dorsal–ventral axis of embryos (Fig. 4a, Supplementary Fig. 4a). Despite a complete loss of detectable MTs, epidermal cells expressing Spastin-GFP still elongated, reaching an aspect ratio that was 92% of comparable cells in control embryos expressing GFP (Fig. 4a,c). This contrasted with the maintenance of the wild-type cell shape/MT alignment correlation when cell shape was perturbed (Fig. 3c). If we use this correlation to predict what would happen if MTs drove cell elongation and the MTs were removed, setting the absence of MTs as equal to completely unaligned MTs (MTSD=90), then the eccentricity should be ∼0.4 (1.09 aspect ratio), rather than the 0.97 (4.11 aspect ratio) observed. Thus, the MTs are clearly not driving the substantial elongation of the cells from their early stage 12 isotropic shapes to the highly elongated shapes at stage 15.

Loss of MTs did however cause a detectable reduction of cell elongation (aspect ratio reduced 25% and eccentricity by 1% (*P*=0.02, Wilcoxon test, Fig. 4a,c). This reduction in cell shape is comparable to that caused by loss of PCP components Fat and Four-Jointed, and is consistent with the similar elongation defects observed when Spastin was expressed in the thinner cell stripes driven by *engrailed::Gal4* (ref. 27). However, this reduction was only significant when we compared with cells in embryos expressing GFP with *paired::Gal4* rather than Spastin-GFP, but not when we compared with adjacent wild-type cells in the same embryo (*P*=0.06, Wilcoxon test, Fig. 4c). This suggested there might be non-autonomy in cell elongation, with cells lacking MTs being elongated by adjacent wild-type cells and vice versa. To test whether cells lacking MTs required adjacent wild-type cells to elongate, we repeated the experiment with a different Gal4 driver to express Spastin-GFP in a block of cells along the anterior–posterior axis of embryos, so that all dorsal cells lacked MTs from onset of elongation (*pannier*::*Gal4*, Supplementary Fig. 4a). As before, the cells underwent most of their elongation and had a similar reduction in elongation (23% aspect ratio, 1% eccentricity, *P*=0.008, Mann–Whitney test, Fig. 4b,d). Thus, MTs do contribute to cell elongation, but are not essential for the bulk of the changes. While MT alignment responded completely to changes in cell shape, cell shape only responded modestly to loss of MTs. This favours a model where cell shape drives MT alignment.

### Cell geometry is sufficient to organize MTs *in silico*

To test whether cell shape could be sufficient to determine the MT organization within a cell and gain understanding of how MTs follow cell shape, we developed a minimal *in silico* model of MT growth in a two-dimensional trapezoid cell representing the sub-apical region. We simulated MT behaviour based on the following initial assumptions. Each MT grows along a straight-line trajectory from a MT ‘seed' and the seeds were uniformly distributed around the cell periphery (in contrast to a PCP mechanism that biases the growth around the periphery), consistent with observed MT regrowth in epidermal cells following depolymerization by colcemid^27^. The MT angle at the seed was chosen randomly from a uniform distribution. MTs were allowed to grow stochastically with constant probabilities of MT polymerization, depolymerization, rescue and catastrophe (see the ‘Methods' section). MTs were not allowed to cross each other, assuming that MTs move in a plane (see the ‘Methods' section). Upon reaching a cell boundary we allowed MTs to stabilize^9^,^28^. We then varied the eccentricity of the cell from 0.7 to 0.95 to match epidermal cells in wild-type embryos (Fig. 2a,b).

**Figure 5:**
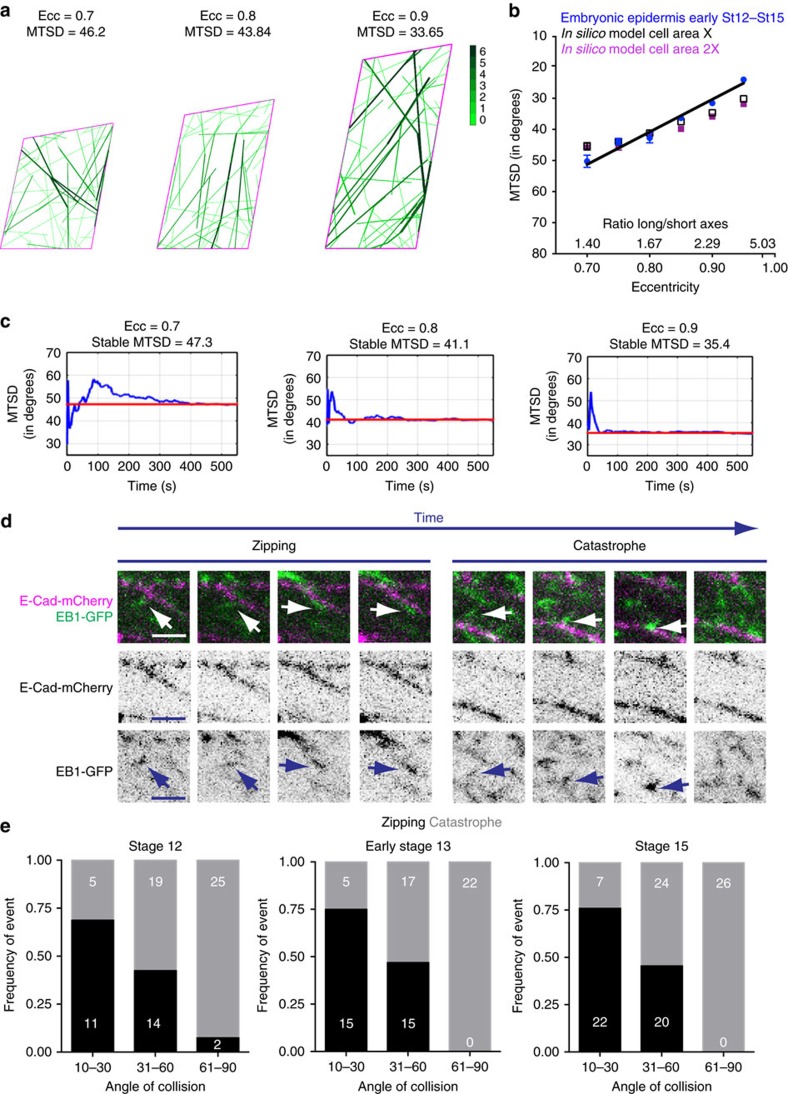
An *in silico* model of MT growth recapitulates MT organization in epithelia and predicts existing angle-dependent outcomes upon MT-cell membrane collisions. (**a**) Example images of *in silico* cells showing that simulated MTs become aligned (MTSD), with larger eccentricities (Ecc); MTs are green, cell apical outline is purple. Colour bar indicates the number of MTs that are bundled due to zipping upon MT–MT or MT–cell boundary collisions. (**b**) The correlation between MTSD and eccentricity of wild-type epidermal cells (St 12—St 15, blue, to be able to compare, cells were selected at the indicated eccentricity±0.01) and *in silico* cells with different eccentricities and apical cell areas (black-X, purple 2X -two fold area increment-) is indistinguishable. Black line depicts shared linear regression for wild-type epidermal and *in silico* cells. Error bars show mean±s.e.m.(**c**) Changes in MTSD with time in single experiments (At *t*=0 MTs are allowed to grow), showing the time required by simulated MTs to reach a stable degree of alignment (that is, MTSD value of convergence) for 0.7, 0.8 and 0.9 cell eccentricities. The red line indicates the convergence MTSD value. (**d**) Sequential images obtained from dorsal–lateral epidermis of live embryos expressing E-Cad-mCherry (purple) and EB1-GFP (green). EB1 comets zip-up with the cell membrane (left) and undergo catastrophe (right). Arrows indicate an EB1 comet undergoing zipping or catastrophe. Scale bars, 2.5 μm. (**e**) Frequency of zipping (black) and catastrophe (grey) events on EB1 comet collisions with the cell membrane in stage 12, early stage 13 and stage 15 embryos. The angle of EB1-GFP comets with respect to the membrane was measured and each event was placed in one of 3 angle categories: 10–30, 31–60 and 61–90 (degrees). Numbers within the graph are observed events per stage. Statistical analysis was performed with two-way ANOVA. Data shown were obtained from 3 embryos and 72 events (stage 12); 4 embryos and 74 events (early stage 13); and 8 embryos and 99 events (stage 15).

Using only these initial assumptions, we found that although simulated MTs became aligned, they oriented along the cell's short axis (Supplementary Movie 2), as observed in plant cells^16^. We therefore adjusted the parameters until we successfully recapitulated the observed MT behaviour. We first tried permitting MTs to cross over each other when they collide, given that they are not limited to such a strict plane as in our model. However, MTs still failed to reach experimentally observed values of MTSD at a particular eccentricity (Fig. 2a,c, Supplementary Movie 3), and instead generated a stable MT mesh. We reasoned this mesh was caused by how we were specifying the consequence of MTs hitting cell boundaries. Therefore, we changed it so that the MTs underwent catastrophe when they contacted a cell boundary (Supplementary Movie 4). This showed some improvement, but the simulated MTs did not form the MT bundles observed *in vivo*, either in fixed (Fig. 2a) or live epidermal cells^27^. Therefore, we used reported angle-dependent outcomes for MT–MT collisions, where depending on to the collision angle MTs either zip-up, cross over or undergo catastrophe^29^, and also applied the same rule to MT–cell boundary collisions. With these modifications, the simulated MTs fit our observed data very well. They formed dynamic aligned bundles that become aligned with the cell's long axis and became increasingly aligned as the cell became more elongated (Fig. 5a and Supplementary Movie 5–8), matching the observed correlation between MTSD and eccentricity in epidermal cells (*P*=0.338, Prism GraphPad shared linear fit of correlation comparison, Fig. 5b). Simulated MTs converged to a stable MTSD value within 110 to 440 s (Fig. 5c), consistent with epidermal MTs regaining their wild-type organization within 1040, s following release from colcemid-induced depolymerization^27^. Additionally, the linear regressions of MTSD-eccentricity in two different *in silico* cell sizes were fit with the same equation (*P*=0.14, Prism GraphPad shared linear fit of correlation comparison, Fig. 5b), consistent with our result with *CyclinA* mutant embryos. Altogether, the *in silico* model of MT growth recapitulates the experimental data from the epidermis, showing that a simple set of rules can translate changes in the shape of the apical cell surface into the observed changes in MT organization, and indicating that a physical mechanism dependent on cell shape changes could dictate the self-organization of sub-apical MTs in the embryonic epidermis. Furthermore, it predicts the occurrence of angle-dependent outcomes of MT–MT and MT–cell boundary collisions in animal epithelia, and suggests they will be a key part of the machinery that determines this morphogenetic change.

To test the prediction of angle-dependent outcomes of MT collisions in the *Drosophila* embryonic epidermis, we examined live embryos expressing EB1-GFP and E-cad-mCherry at stages 12, 13 and 15. This enabled us to examine collisions between the growing MTs and the cell borders. We were not able to generate suitable imaging conditions to visualize collisions of growing MTs with the sides of existing MTs, due to small cell size and high MT density. At all stages we found examples of EB1 comets either zipping-up with the cell membrane or undergoing catastrophe (Fig. 5d, Supplementary Movie 9). Consistent with observations of MT–MT collisions in plant cells^30^, and the importance of introducing this factor into our model, the outcome correlated with the angle at which the MTs approach the cell border. Zipping was most frequent for angles <30° (Fig. 5d,e), whilst catastrophes were most frequent for angles >60° (Fig. 5d,e). The correlation between the outcome and angle was the same at all stages examined (*P*=0.95 for both zipping and catastrophe, two-way ANOVA, Fig. 5e). We did not observe planar polarized differences in angle-dependent outcomes of MT–cell boundary collisions, suggesting that they are either caused purely by physical mechanisms, or by factors that are uniformly distributed around cell periphery. The similar degree of correlation in *Drosophila* and plant cells suggests a conserved mechanism. Thus, the confirmation of angle-dependent outcomes of collisions between MTs and cell boundaries in *Drosophila* embryonic epidermal cells supports the importance of this variable in our *in silico* model.

**Figure 6:**
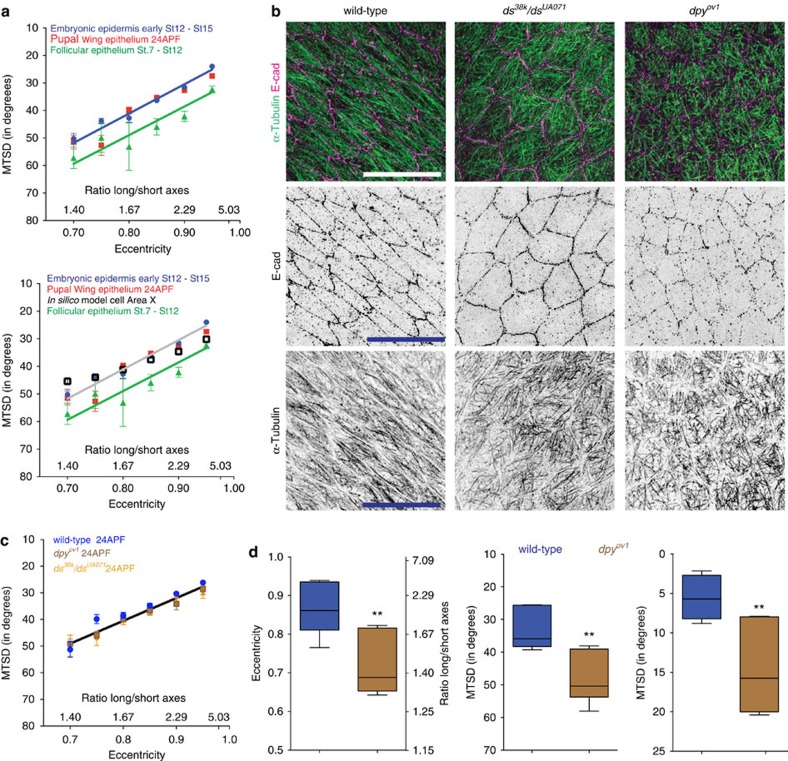
Cell shape-dependent mechanisms of MT organization are widespread in *Drosophila* epithelia. (**a**) MTSD versus elongation correlations are indistinguishable in the embryonic epidermis (St 12—St 15, blue, both graphs), pupal wing epithelium (24APF, red, both graphs) and *in silico* cells (F, black, bottom only). In the follicular epithelium (St 7—St12 egg chambers, green, both graphs), although the slope is retained, the correlation is shifted towards larger MTSD values. Bottom graph shows top graph data plus *in silico* cells. (**b**) Representative images of pupal wing epithelium 24APF, visualized as described in Fig. 1, from wild-type (left), pupal wings from transheterozygous *ds*^*38k*^/*ds*^*UA071*^ mutants (centre) and pupal wings from *dpy*^*ov1*^ mutants (right). Scale bars, 10 μm. (**c**) MTSD versus eccentricity correlation is retained between wild-type (blue), *ds*^*38k*^/*ds*^*UA071*^ (orange) and *dpy*^*ov1*^ (brown) 24APF pupal wing epithelium cells. (**d**) Quantification of cell elongation (eccentricity and cell's long/short axes ratios; left), MTSD (centre) and MTDEV (right) shows reduced cell elongation and MT organization in *dpy*^*ov1*^ pupal wings. Cells from 7 pupal wings (three independent experiments) were analysed. Statistical analysis was performed with Mann–Whitney test (eccentricity) and unpaired *t*-test (MTSD and MTDEV). Asterisks represent statistical significance P**<0.01. Each dot in **a** and **c** represents the mean MTSD value (mean±s.e.m.) of experimental or simulated cells with corresponding eccentricities±0.01. Cell number and corresponding eccentricity (parenthesis) were in **a**: for embryonic epidermis: 100 (0.7), 138 (0.75), 182 (0.8), 245 (0.85), 334 (0.9), 520 (0.95) from 42 wild-type embryos; for pupal wing epithelium: 35 (0.7), 35 (0.75), 45 (0.8), 43 (0.85), 46 (0.9), 35 (0.95) from 4 wild-type pupae; for follicular epithelium: 31 (0.7), 15 (0.75), 11 (0.8), 19 (0.85), 16 (0.9), 13 (0.95) from 6 wild-type female flies. In **d**, for wild-type: 22 (0.7), 26 (0.75), 42 (0.8), 60 (0.85), 94 (0.9), 109 (0.95) from 7 pupae; for *dpy*^*ov1*^: 62 (0.7), 82 (0.75), 61 (0.8), 51 (0.85), 43 (0.9), 9 (0.95) from 7 pupae; for *ds*^*38k*^/*ds*^*UA071*^: 24 (0.7), 32 (0.75), 54 (0.8), 45 (0.85), 37 (0.9), 9 (0.95) from 4 pupae.

To test if cell shape predicts MT organization only in epidermal cells at a particular stage of development, or also in other epithelia, we examined the MTSD-eccentricity correlation in *Drosophila* pupal wing and follicular epithelia. Sub-apical MTs from wing epithelium of pupae at 24 h after puparium formation (24APF) and basal MTs in the follicular epithelium from stage 7 to 12 egg chambers displayed linear correlations with cell eccentricity (Fig. 6a). The MTSD-eccentricity correlations in the epidermis, pupal wing epithelium and *in silico* model were fit with the same linear regression equation (*P*=0.18, Prism GraphPad shared linear fit of correlation comparison, Fig. 6a). In contrast, the MTSD-eccentricity correlation in the follicular epithelium showed a shift to higher MTSD values throughout all eccentricities (Fig. 6a), suggesting that despite the important role of cell shape, additional mechanisms might be involved in the alignment of MTs in this tissue.

Given the same linear dependency between MTSD and cell shape in embryonic epidermis and pupal wings, we sought to test whether cell shape changes are also required for MT organization in pupal wings. Similarly to the embryonic epidermis, evidence suggested that the PCP pathway regulates MT organization in the pupal wing^11^. We checked whether perturbation of the PCP pathway in the pupal wing affects both cell shape and MT organization, as we had found in the embryo. In pupal wings at 24APF the cells were elongated and MTs were aligned and oriented parallel to the cell's long axis (Fig. 6b). We analysed *dachsous* transheterozygous mutants (*ds*^*38k*^/*ds*^*UA071*^, Fig. 6b), which were previously reported to disrupt MT organization in 24APF pupal wings^11^, and detected an increase in MTSD and MTDEV (19 and 72% increase, respectively, *P*<0.0001 for both, Mann–Whitney test, Supplementary Fig. 4b), consistent with the previous work^11^. However, this increase was accompanied by a reduction in cell elongation (17% decrease in aspect ratio, 9% reduction in cell eccentricity, *P*<0.0001, Mann–Whitney test, Supplementary Fig. 4b). Moreover, the linear regression between MTSD and eccentricity remained the same as wild type (*P*=0.14, Prism GraphPad shared linear fit of correlation comparison, Fig. 6c). Thus, perturbation of PCP pathway in the embryonic epidermis (Fig. 1a,b) and pupal wing epithelium affected both cell shape and MT organization.

Finally, we examined whether changes to the shape of these cells also has an impact on MT organization. We perturbed their shape using a hypomorphic mutant in the gene encoding the apical extracellular-matrix (aECM) protein Dumpy (*dpy*^*ov1*^), which connects the apical surface to the overlying aECM (ref. 31). In *dpy*^*ov1*^ mutants there is a failure in wing blade cell elongation, which is clearest at 24APF (ref. 32; Fig. 6b,d). The reduced cell elongation (29% decrease in aspect ratio, 18% reduction in eccentricity, corresponding to *P*=0.007, Mann–Whitney test, Fig. 6b,d) was accompanied by reduced MT alignment, as revealed by the increase in MTSD and MTDEV (48% increase, *P*=0.001 and 170% increase, *P*=0.002 respectively, unpaired *t*-test, Fig. 6b,d). Morever, the correlation between MTSD and eccentricity was retained between wild-type and *dpy*^*ov1*^ (*P*=0.16, Prism GraphPad shared linear fit of correlation comparison, Fig. 6c), showing that the observed reduction in MT alignment in *dpy*^*ov1*^ is proportional to the reduction of cell elongation. Thus, combining these results with those in the embryo, we have perturbed epithelial cell shape in three different ways, and in each case the level of MT alignment matched the cell shape, and the observed effects exceeded that of PCP mutants. Whilst we cannot rule out the possibility that the experimental approaches we have used to alter cell shape somehow alter PCP mechanisms, our results fit with the simplest model, which is that the response of MTs to the collision with the cell periphery is sufficient for their alignment in response to cell elongation.

## Discussion

Here, we report evidence for a physical, cell shape-dependent mechanism controlling MT organization within cells in an intact epithelium. Our experimental perturbations of cell elongation had a greater impact on MT organization than perturbations of MTs had on cell shape. Using numerical simulations we were able to recapitulate the observed MT organization in a purely geometrical model and predicted that cell shape is translated into MT organization through MT self-organizing processes not previously described in animal epithelia. This prediction is further supported by our finding that one of the key aspects of MT behaviour suggested to be required for MT self-organization by our *in silico* model, namely angle-dependent outcomes of MT collisions with cell boundaries, could be documented in *Drosophila* embryonic epidermal cells.

A physical cell shape-dependent mechanism of MT organization could provide a simple, fast and cost-effective mechanism to coordinate cell shape changes with the cell functions that require a particular MT organization within the cell. Thus, we suggest that physical adaptation of MTs to cell geometry is a robust mechanism to couple epithelial morphogenetic events with the asymmetric distribution of proteins at the cell membrane. In *Drosophila*, changes in the overall shape of the embryo lead to elongation of the epidermal cells, which in turn, may be sufficient to determine the formation of a parallel array of MTs aligned with the cell's long axis. In the *Drosophila* epidermis, the asymmetric distribution of E-cadherin, which requires the aligned organization of sub-apical MTs, is important for tissue organization^4^. Therefore, it is the epidermal cell shape morphogenesis that sets the initial conditions for the asymmetric distribution of E-cadherin, which eventually feeds back to fine tune epidermal tissue organization. We hypothesize that a similar mechanism could drive the planar polarized distribution of Frizzled and Dishevelled, or other core PCP components, in the wing epithelium^13^,^33^, by coupling morphogenesis of the wing epithelial cells^34^ with physical cell shape-dependent alignment of the sub-apical MTs.

Physical and signalling mechanisms may act together with differing emphasis to direct MT organization, depending on the tissue and developmental cues. We hypothesize that physical adaptation of MTs to cell geometry will generally direct MT self-organization in eukaryotes unless a signalling pathway overcomes this default pattern. This is supported by MTSD-eccentricity correlation in different *Drosophila* epithelial tissues, our *in silico* model, and growth of MTs *in vitro* in elongated microchambers, which results in spontaneous formation of aligned MT arrays parallel to the long axis of the chamber^35^. Our *in silico* model predicts that angle-dependent MT–cell boundary outcomes contribute to MT alignment, and we confirmed the occurrence of angle-dependent MT–cell boundary collision outcomes in the *Drosophila* epidermis. The question remains whether the critical angle at which MTs undergo zipping or catastrophe upon collision results purely from biophysical properties of MTs or is actively controlled, for example, by MT-binding proteins. However, comparison of MT response to cell shape changes in *Drosophila* and yeast (our work, ref. 5), and the similarity of angle-dependent MT collision outcomes in such diverse organisms as *Drosophila* and plants (our work, ref. 30) suggest that the physical alignment of MTs is a widespread mechanism.

Although cell shape determines MT organization in epithelia, the opposite is not true and MTs were not essential for the majority of epidermal cell elongation. This is in contrast to the importance of MTs for both changing and maintaining cell shape of individual cells in culture^6^,^36^,^37^. This discrepancy in the role of MTs might reside in the context: a cell integrated in an epithelial tissue adheres to the neighbouring cells, and is subject to forces that can increase the rigidity of the cell cortex, thus limiting the effect of MTs on cell shape.

In summary, we demonstrated that cell shape determines MT organization in *Drosophila* epithelial tissues. Based on our findings, we propose a model in which MTs self-organize by adapting to cell shape through the intrinsic dynamic instability of MTs, and their collision behaviour with other MTs and the cell boundaries, and validated the feasibility of this model by computational simulation.

## Methods

### Fly stocks

*w^bf^f^5^*, *w^1118^, ft^G-rv^*, *Df(2L)BSC217, ds^38k^*, *fj^d1^*, *fz^21^*, *stan^e59^*, *ush^1^*, *paired::Gal4, pannier::Gal4, UAS::CD8-mCherry*, (Bloomington, 157, 3605, 1894, 9694, 288, 6373, 41787, 41776, 35503, 1947, 25758, 27392, respectively), *Gal4^332.3^* (ref. 24), *UAS::Spastin-GFP* (ref. 26; provided by S. Noselli), *UAS::GFP, ds^UA071^* (both provided by J. Casal), *UAS::reaper* (ref. 25), *traffic jam-Gal4* and *UAS::myr-GFP, dpy^ov1^* (ref. 32), *EB1-GFP* (ref. 4), *E-Cad-mCherry* (ref. 38).

### Immunostaining and live imaging procedures

Egg collections were done for 1 h at 25 °C, except for the experiments in Fig. 3, which were 1.5 h collections. Gal4 driven expression was performed at 25 °C. Embryos were dechorionated in 75% bleach for 3 min. To visualize MTs in the embryonic epidermis, a formaldehyde–methanol sequential fixation was performed as follows: dechorionated embryos were fixed in 1:1 10% formaldehyde (methanol free, #18814, Polysciences Inc.) in PBS:heptane for 20 min at room temperature (RT) and then fixed/devitellinized for 45 s in 1:1 ice-cold methanol:heptane. Finally, embryos were washed three times in ice-cold methanol, kept in methanol between 6 and 24 h at −20°, rehydrated in 1:1 PBS with 0.3% Triton X-100 (T9284, Sigma): methanol and washed one time in PBS with 0.3% Triton X-100.

For live imaging, embryos were dechorionated in 50% bleach for 4 min, and mounted on a 35 mm Petri dish with Voltalef Oil 10 s and imaged immediately.

Pupal wings were dissected from pupae aged 24 h after puparium formation at 25 °C. Pupae were removed from the puparium and fixed for 2 h in 10% formaldehyde (methanol free, #18814, Polysciences Inc.) in PBS. Next, pupal wings were dissected in PBS and washed three times in PBS with 0.3% Triton X-100 (T9284, Sigma).

Ovaries were dissected from flies aged 48 h at 25 °C in S2 medium (PromoCell) at RT, fixed for 20 min in 10% formaldehyde (methanol free, #18814, Polysciences Inc.) in PBS, and washed three times in PBS with 0.1% Triton X-100 (T9284, Sigma).

Staining procedures were performed as follows: rehydrated embryos and fixed pupal wings were blocked for 2 h in 5% Native Goat Serum (ab7481, Abcam) in PBS with 0.3% Triton X-100. Fixed ovaries were blocked for 1 h in 0.1% bovine serum albumin. Primary antibody incubations were done overnight at 4 °C. Primary antibodies used were mouse anti-α-Tubulin 1:1,000 (T6199, Sigma), rat anti-E-cadherin 1:50 (DCAD2, Developmental Studies Hybridoma Bank), and rabbit anti-GFP 1:1,000 (ab290, Abcam). Incubation with secondary antibody was performed for 2 h at 25 °C. Alexa Fluor fluorophore (Alexa Fluor 405, 488, 568, 594 and 647)-coupled secondary antibodies were used in 1:50 (405, Abcam) or 1:300 (488, 568, 594 and 647, Jackson ImmunoResearch). Finally, embryos were mounted in Vectashield (Vector Laboratories).

### Image acquisition

All images from fixed samples were acquired at RT (20–22 °C) using a super resolution Deltavision OMX 3D-SIM (3D-SIM) V3 BLAZE from Applied Precision (a GE Healthcare company), with the exception of Fig. 4, Supplementary Fig. 3a and Supplementary Fig. 4a, that were acquired with an Olympus FV1000 upright confocal. Deltavision OMX 3D-SIM System V3 BLAZE is equipped with 3 sCMOS cameras, 405, 488 and 592.5 nm diode laser illumination, an Olympus Plan Apo N × 60 1.42 numerical aperture (NA) oil objective, and standard excitation and emission filter sets. Imaging of each channel was done sequentially using three angles and five phase shifts of the illumination pattern. The refractive index of the immersion oil (Cargille) was adjusted to either 1.513 or 1.516 (depending on the emission wavelength of the fluorophore used to detect the MTs) to minimize the optical effect of spherical aberration, which is caused by mismatches between the refractive index of the lens, immersion medium and the specimen mounting medium.

Sections were acquired at 0.125 μm z steps. Raw OMX data were reconstructed and channel registered in SoftWoRx software version 5.5 (Applied Precision, a GE Healthcare company).

For Fig. 4, Supplementary Fig. 3a and Supplementary Fig. 4a, images were acquired on Olympus FV1000 upright confocal microscope using × 20 (0.75 NA, air), × 40 (1.3 NA, oil) and × 100 (1.40 NA, oil) UPlanSApo objectives. For Fig. 4, a single stack of 4–11 z-sections spaced by 0.3 μm was imaged from each embryo. 16-bit depth images were taken at a magnification of 0.12 μm pixel^−1^ using the × 100 objective. Images were maximum intensity projected along the sub-apical domain, which was determined using E-cadherin junctional signal as a reference. For Supplementary Fig. 3a, single sections were taken at a magnification of 0.52 μm pixel^−1^ with the exception of the *ush*^*1*^ embryo that was taken at 0.44 μm pixel^−1^ using the × 20 objective. For Supplementary Fig. 4a, single sections were taken at a magnification of 0.31 μm pixel^−1^ using the × 40 objective. Image acquisition was done with FV10-ASW software for Olympus FV1000.

Live time-lapse imaging was acquired with an Olympus IX81 Spinning Disk using a × 100 (1.40 NA, oil) UPlanSApo objective+ × 1.6 internal objective at room temperature (20–22 °C). To analyse MT organization (EB1 trajectories, Fig. 2e and Supplementary Movie 1, time-lapse sequences consisted of 16-bit images at a magnification of 0.105 μm pixel^−1^, each time point containing a projection of 2 sections spaced in z by 0.3 μm, at 6.3 s time intervals. To analyse the angle-dependent outcomes of MT-cell boundary collisions, time-lapse sequences consisted of 16-bit images at a single plane with a time interval of 2.65 s and a magnification of 0.105 μm pixel^−1^.

Images were maximum intensity projected using Fiji (www.fiji.sc). Figures were assembled using Adobe CS3 Photoshop and Illustrator (www.adobe.com). Processing of images shown in figures involved adjusting of gamma settings.

### Automated image analysis

OMX 3D-SIM stacks corresponding to the sub-apical domain of the cells were maximum (max) intensity projected. The sub-apical domain of the cell was determined using the E-cadherin junctional signal as a reference for its basal limit, and the absence of α-Tubulin signal as a reference for its apical limit. In all analyses conducted on dorso-lateral epidermal cells, we excluded the leading edge (first row), given its differential identity compared with the rest of the dorso-lateral epidermal cells.

To analyse cell shape and MT organization on a cell-by-cell basis we developed a script in MATLAB R2014b (Mathworks, www.mathworks.co.uk). E-cadherin signal from a max intensity projection was used to obtain cell outlines using Packing Analyser V 2.0 software^34^ as shown in Supplementary Fig. 1a–c. Then, the cell outlines were used to identify each cell as an individual object and fit it to an ellipse to calculate eccentricity, the lengths of major and minor axes of the ellipse, and aspect ratio (referred as cell's long and short axes, Supplementary Fig. 1c), and the direction of the ellipse major axis (cell orientation).

To analyse MT organisation, the α-Tubulin signal levels from max intensity projected images were adjusted such that 0.5% of the data with lowest intensities were set to black and the 0.5% of the data with highest intensities were set to white to increase the contrast. Next, the α-Tubulin signal within each cell was filtered using the cell outline as a mask (Supplementary Fig. 1c) and the magnitude of the signal according to its direction (that is, gradient of the signal) analysed by convolving the filtered α-Tubulin signal with two 5 × 5 Sobel operators, G_*x*_ and G_*y*_, (Supplementary Table 1), corresponding to the *x* and *y* coordinates of the image respectively.

The resulting gradient approximations for *x* (G_*x*_) and *y* (G_*y*_) coordinates were combined to obtain the α-Tubulin magnitude gradient (*M*) and direction gradient (*D*):


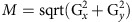



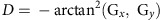


*M* and *D* were integrated into a matrix to assign each pixel a direction and magnitude of change in the intensity of α-Tubulin signal. To reduce the noise, we discarded those pixels in which the magnitude was <22% of the maximum magnitude of change within each cell. The remaining pixels were binned with respect to their direction gradient (bin size=4°), and the magnitude of change corresponding to each bin was normalized to the total magnitude to build a histogram of the relative magnitude of change in α-Tubulin signal with respect to its direction. Given that Sobel operators identify the edges (or direction of change) of the signal in an image, we applied a 90° correction to the binned gradient directions to align the histogram with direction of the α-Tubulin signal.

The resulting histograms of magnitude of α-Tubulin signal changes with respect to its direction were fit with the Von Mises distribution in MATLAB^39^. The estimated mean of the fitted curve was used as the main direction of the α-Tubulin signal (μ, MT Main Direction, Supplementary Fig. 1d), and its s.d. (*σ*, Supplementary Fig. 1d) was used as a measure of MT alignment (MT s.d., MTSD, Supplementary Fig. 1d). To measure the degree of alignment of the MT array to the cell's long axis, we calculated the absolute difference between the main direction of the MTs and the cell orientation (MTDEV, Supplementary Fig. 1d).

To analyse EB1-GFP trajectories alignment, the images corresponding to 10 sequential time points (56.7 s length) were analysed separately as described for fixed embryos using the Matlab script.

For validation with Fiji Directionality tool (http://imagej.net/Directionality), we selected the Fourier Components option to compare with the 5 × 5 Sobel filter in the Matlab script. We analysed the entire field of α-Tubulin staining of stage 12 and 15 embryonic epidermis with Fiji Directionality tool and the Matlab script and compared them to the cell-by-cell analyses of the same images. 7 images for each stage were analysed.

For validation using simulated data, we generated lines (MTs) running through 100 randomly selected points within a simulated cell, at angles that were randomly sampled from normal distributions with s.d.'s of 22, 30 and 40. The eccentricities of simulated cells were 0.7, 0.8, 0.92 and 0.98. To incorporate a decrease in MTSD with increase in eccentricity observed both in experimental and *in silico* data, we have chosen four combinations of eccentricity and MT angles (Supplementary Table 2). Using these figures we produced three images of simulated data for each combination, each containing lines (MTs) with known orientations and lengths. From these images we built histograms of simulated MT length according to the angle and fit these histograms with a Von Mises distribution to obtain a MTSD value based on the simulated data. Simultaneously, these images were analysed with the Matlab script to obtain MTSD values for these simulated MTs.

Matlab script used to analyse MT organization is available from the authors on request.

### Quantification of MT-cell boundary collisions

To quantify the collision angles, we measured the angle formed between the E-Cad-mCherry signal, which was used as a physical reference, and the EB1-GFP comet at the time point preceding the collision against the membrane. We could not resolve collision angles <10°, and therefore, we quantified the collisions by placing the events in one of these 3 categories: 10–30, 31–60 or 61–90. Zipping events were considered as such when the EB1-GFP comet aligned with the cell boundary after the collision for at least one time point. Catastrophe events were considered as such when the EB1-GFP signal was undetectable after collision with the cell membrane.

### *In silico* model of MT growth

We model MTs as one-dimensional polymers composed of Tubulin dimer units^40^ that are constrained to be in a two-dimensional sub-apical domain of a cell and grow in a straight-line trajectory. To set-up the geometry of the two-dimensional cell's sub-apical domain, we set the shortest cell's width to 492 nm (equivalent to the length of 60 Tubulin dimers) and varied the length of the cell's long axis to match the desired eccentricity values. The eccentricities of the cells were 0.7, 0.75, 0.8, 0.85, 0.9 and 0.95, which correspond to cell areas in the range between 0.30 μm^2^ and 1.57 μm^2^. In the second set of experiments with larger cells, we set the shortest cell width to 984 nm and varied the eccentricities between 0.7 and 0.95, corresponding to cell areas between 1.20 and 6.20 μm^2^.

For the time evolution of each MT we used the continuous time Markov Chain model^40^. Each one-dimensional MT can be in one of the two states: either polymerizing or depolymerizing (Supplementary Fig. 5). If a MT is n Tubulin dimers long and is in the polymerizing state, which we denote by A_n_, it adds another dimer with the rate of polymerization (*α*), or changes state to depolymerizing and loses a Tubulin dimer with the rate of catastrophe (*β*′). A MT that is n Tubulin dimers long and in a depolymerizing state, which we denote by B_n_, can either add a dimer with a rescue rate (*α*′), or depolymerize further with the high depolymerization rate (*β*).

All rates (*α*, *α*′, *β*, *β*′) were taken to be constant, assuming that Tubulin concentration did not change significantly during the experiment. To find these rates, we do the following. We set the non-dimensional polymerization rate in the MC model to be *α*=1,000. This corresponds to the dimensional MT growth speed in the model equal to *α* × (1 dimer) × (8.2 nm dimer^−1^) × *R*, where *R* is the dimensional rate in units of s^−1^. Setting this equal to the observed experimental growth speed of MTs in the dorsal–lateral embryonic epidermis (the experimental rate^4^ was 0.15 μm s^−1^), we find *R*=0.01829, s^−1^. The other rates were not available experimentally, so we set them arbitrarily based on the following three facts: first, depolymerization rate is known to be much larger than the polymerization rate; second, MTs are observed to usually grow until they reach the cell boundary or another MT and almost never experience collapse independent of these factors, hence the catastrophe rate was chosen to be much smaller than that of polymerization and depolymerization; and third, the probability of rescue is much higher than that of catastrophe, consistent with previous reports^8^. In summary, the Markov Chain transition rates were polymerization (*α*=1,000), depolymerization (*β*=3,500), rescue (*α*′=4) and catastrophe (*β*′=1). Multiplying these non-dimensional transition rates by the factor *R* one can recover the corresponding dimensional rates (*α*_dimensional_=18.29 s^−1^, *α*′_dimensional_=0.07316, s^−1^, *β*_dimensional_=64.02 s^−1^ and *β*′_dimensional_=0.01829, s^−1^).

We prescribed a fixed number (*n*=220) of MT ‘seeds' (that is, minus ends) for all the experiments. They were distributed uniformly along the perimeter of the cell, as experimentally observed^27^. At the start of each experiment all MT lengths were set to zero.

We imposed the following simple geometric rules for MT–MT and MT–cell boundary interactions. When any two MTs collide, there are three potential outcomes: crossing, zipping and catastrophe^30^. These scenarios are realized depending on the angle of MT–MT collision and on the prescribed probability of catastrophe. If the crossing angle is larger than the critical angle (*θ*_critical_) of 30°, the MT experiences a catastrophe with the prescribed probability *p*_cat_ (*p*_cat_=0.01), and crosses the MT with the probability (1−*p*_cat_). If the crossing angle (*θ*) was <30° (*θ*_critical_), then the probability of catastrophe is *P*=*θ*/*θ*_critical_ × *p*_cat_ and the probability of zipping is (1−*P*). When a MT collides with a cell wall and the angle smaller than *θ*_critical_, the rules for zipping are the same as in the MT–MT collisions. However, if the collision angle is bigger than *θ*_critical_, the MT undergoes a catastrophe.

To estimate the degree of alignment of simulated MTs and the relevant statistics, all the experiments were run until non-dimensional time 10, which corresponds to 550 s. The expected value of MT angles was computed by length-averaging the last 2.5 non-dimensional time units, which corresponds to 250 time points. The resulting data of MT directions was length averaged and binned in 180 bins for each degree of possible MT direction to build a histogram of average MT length with respect to its direction. Next, the histogram was fit using the Von Mises distribution to preserve the consistency between the experimental and simulated analysis of MT organization. Finally, the s.d. of the Von Mises fit was estimated to calculate the MTSD of simulated MTs. Each data point in the MTSD versus Eccentricity plot of simulated MTs is the mean of three runs (simulation experiments).

The *in silico* computer model of MT growth is available from the authors upon request.

### Statistical analysis

Statistical analysis was performed in Graphpad Prism 6.0c (www.graphpad.com). Samples from independent experiments corresponding to each genotype and/or developmental stage (embryos/pupal wings/egg chambers) were pooled and tested for normality with the Shapiro–Wilkinson test. The criteria to select the statistical test was based on the number of groups that are being compared (two or more than two), the control and treatment groups relation (whether they are in the same experiment unit or not) and the result of the normality test, which defined the use of a parametric or non-parametric test. All tests used in this manuscript were based on *t*-test and ANOVA methods. No statistical method to define sample size was used. We set a minimum sample size of four individuals (embryo/pupae) and 300 cells. To estimate the variation in the sample we used s.d. or s.e., and the type of measure was stated in the figure legends when used. In cases when s.d. or s.e. was not used, we showed the distribution of samples within groups using a Box–Whiskers plot. The centre line of each plot indicates the median, while the top and bottom of each box indicate the third and first quartiles, respectively. Top and bottom whiskers denote the maximum and minimum values, respectively. No randomization nor blinding method was applied to the experimental design. The analysis of the deviation from linearity and exponential one-phase decay fits was performed in MATLAB R2014b (www.mathworks.co.uk).

### Data availability

The data that support the findings of this study including source data for figures and Matlab scripts are available from the corresponding authors upon request.

## Additional information

**How to cite this article:** Gomez, J. M. *et al.* Microtubule organization is determined by the shape of epithelial cells. *Nat. Commun.* **7,** 13172 doi: 10.1038/ncomms13172 (2016).

## Supplementary Material

Supplementary Information Supplementary Figures 1-5, Supplementary Tables 1-2.

Supplementary Movie 1 Time-lapse of a representative epidermal cell from Stage 12 (a), Early Stage 13 (b) and Stage 15 (c) embryo over a course of 56.7 sec (frame time interval = 6.3 sec). EB1-GFP comets are in green (single channel in gray, center panel) and shg-Cherry (E-cad) is in red (single channel in gray, right panel). MTSD and eccentricity values for each time-point are depicted in blue in upper-right corner of each single channel. Scale bar - 5μm

Supplementary Movie 2 Simulated growth of MTs in a cell with eccentricity=0.95, in case when MTs are not allowed to crossover each other. MTs undergo catastrophe upon MT-MT collisions and become stabilized at the cell boundary upon MT-cell boundary collisions. MTs are in green and cell boundary in purple. Colour bar indicates the number of MTs in a MT bundle.

Supplementary Movie 3 Simulated growth of MTs in a cell with eccentricity=0.95, in case when MTs are allowed to undergo crossover and become stabilized at the cell boundary upon MT-cell boundary collisions, but are not allowed to zip-up upon MT-MT collisions. MTs are in green and cell boundary in purple. Colour bar indicates the number of MTs in a MT bundle.

Supplementary Movie 4 Simulated growth of MTs in a cell with eccentricity=0.95, in case when MTs are allowed to crossover and undergo catastrophe upon MT-cell boundary collisions, but MTs are not allowed to zip-up upon MT-MT collisions. MTs are in green and cell boundary in purple. Colour bar indicates the number of MTs in a MT bundle.

Supplementary Movie 5 Simulated growth of MTs in a cell with eccentricity=0.7, in case when angle-dependent outcomes of MT-MT and MT-cell boundary collisions are introduced. MTs are in green and cell boundary in purple. Colour bar indicates the number of MTs in a MT bundle.

Supplementary Movie 6 Simulated growth of MTs in a cell with eccentricity=0.8, in case when angle-dependent outcomes of MT-MT and MT-cell boundary collisions are introduced. MTs are in green and cell boundary in purple. Colour bar indicates the number of MTs in a MT bundle.

Supplementary Movie 7 Simulated growth of MTs in a cell with eccentricity=0.9, in case when angle-dependent outcomes of MT-MT and MT-cell boundary collisions are introduced. MTs are in green and cell boundary in purple. Colour bar indicates the number of MTs in a MT bundle.

Supplementary Movie 8 Simulated growth of MTs in a cell with eccentricity=0.95, in case when angle-dependent outcomes of MT-MT and MT-cell boundary collisions are introduced. MTs are in green and cell boundary in purple. Colour bar indicates the number of MTs in a MT bundle.

Supplementary Movie 9 Time-lapse of a representative MT-Cell boundary zipping (arrowheads) and catastrophe (arrows) events in epidermal cells from Stage 12 (a), Early Stage 13 (b) and Stage 15 (c) embryos over a course of 16.2 sec (frame time interval = 2.7 sec). EB1-GFP comets are in green and shg-Cherry (E-cad) is in red. Scale bar - 5μm
