# Supplementary material for: Microtubule organization is determined by the shape of epithelial cells
Source: Nat Commun. 2016 Oct 25;7:13172. doi: 10.1038/ncomms13172 (PMC5093320; doi:10.1038/ncomms13172)
Supplement: Supplementary Information — Supplementary Figures 1-5, Supplementary Tables 1-2. [file ncomms13172-s1.pdf]

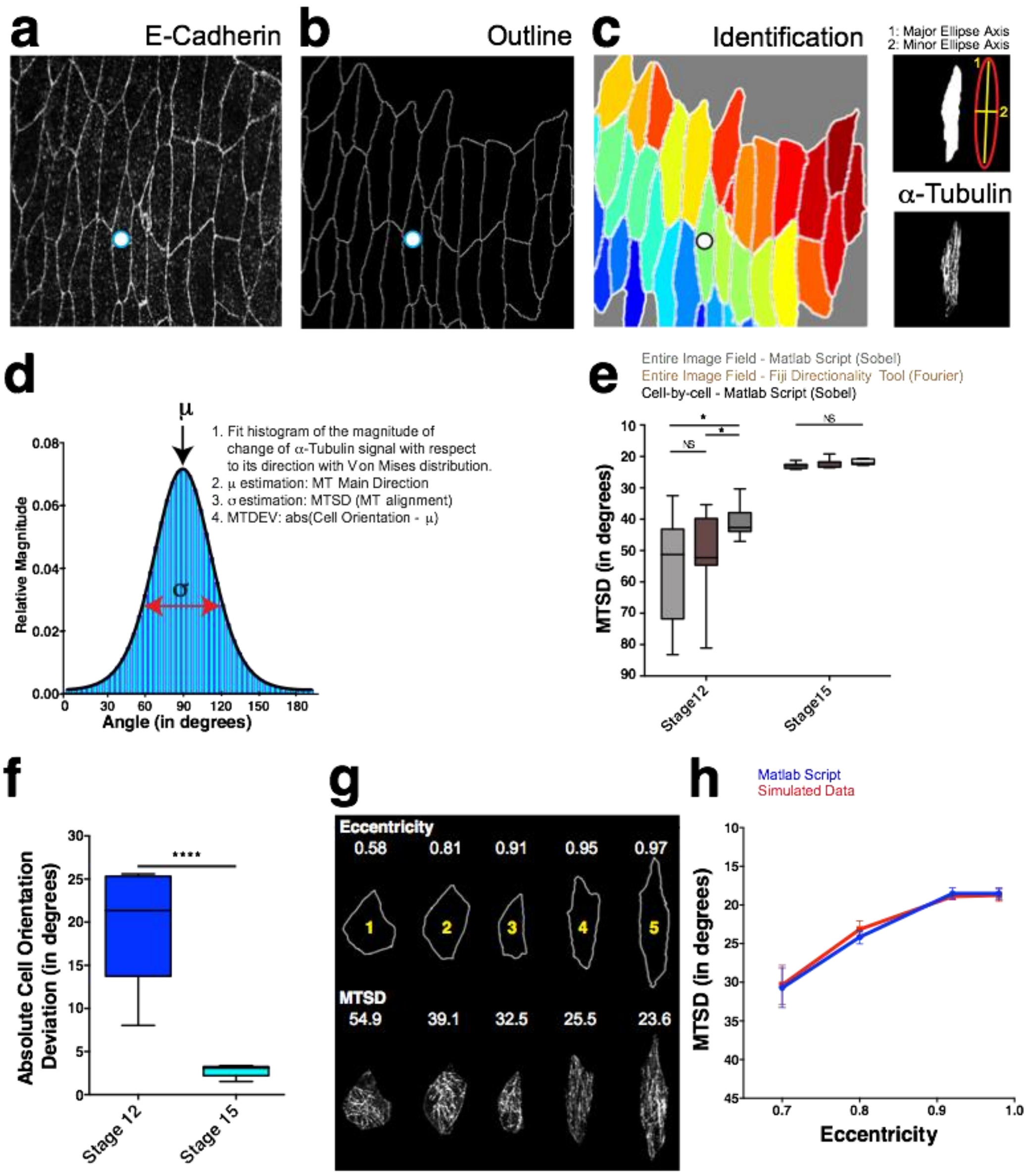

Supplementary Figure 1. Automated image analysis of cell shape and microtubule organization. (a-d) Strategy of cell-by-cell analysis of cell shape and MTs organization. A dot marks the same cell at each step. **(a)** Example image of dorso-lateral epidermis of a stage 14 embryo with cell outlines marked by staining E-

cadherin. **(b)** Cell outline image generated from (a) with Packing Analyzer V2.0 software[34]. **(c)** Cells were identified as different objects, fit to an ellipse and the eccentricity of the ellipse and the orientation and length of the ellipse's major and minor axes was measured (upper-right), and the MTs within each cell, visualized by α-Tubulin staining, were extracted (lower right). **(d)** Histogram of the fraction of α-Tubulin signal within a cell oriented in each direction (see Methods), fitted with Von Mises distribution. From the Von Mises Fit μ (mean) and σ (standard deviation), MT organization parameters (MTSD, MTDEV) were estimated. (**e**) Independent validation of Matlab script cell-by-cell method by comparison with Matlab script and Fiji Directionality tool Fourier-transform analyses of entire cell field of α-Tubulin signal. In Stage 15, no significant differences in MTSD measured with three methods were detected. Statistical comparison per stage was performed with paired one-way ANOVA and Fisher's LSD post-hoc analysis. * - P<0.5, NS - not significantly different. (**f**) Absolute standard deviation of cell orientation (0º-90º) of cells from images in F, showed a larger deviation in Stage 12. Statistical comparison was performed with unpaired t test. **** , P< 0.0001. In e-f, 7 images corresponding to 7 embryos per stage were analyzed. (**g**) Image containing increasingly elongated cell outlines and the sub-apical MTs within each of these cells, with the corresponding eccentricity and MTSD values. (**h**) Matlab Script validation with simulated data. Images with simulated MTs sampled from normal distributions with standard deviations of 40 (Eccentricity=0.7), 30 (Eccentricity=0.8) and 22 (Eccentricity=0.92 and 0.98), were analyzed with the Matlab Script and compared to MTSD obtained by fitting with Von Mises the histogram of simulated MTs lengths vs. direction. Each datapoint is the mean ± s.d. of 3 simulated data images per eccentricity value.

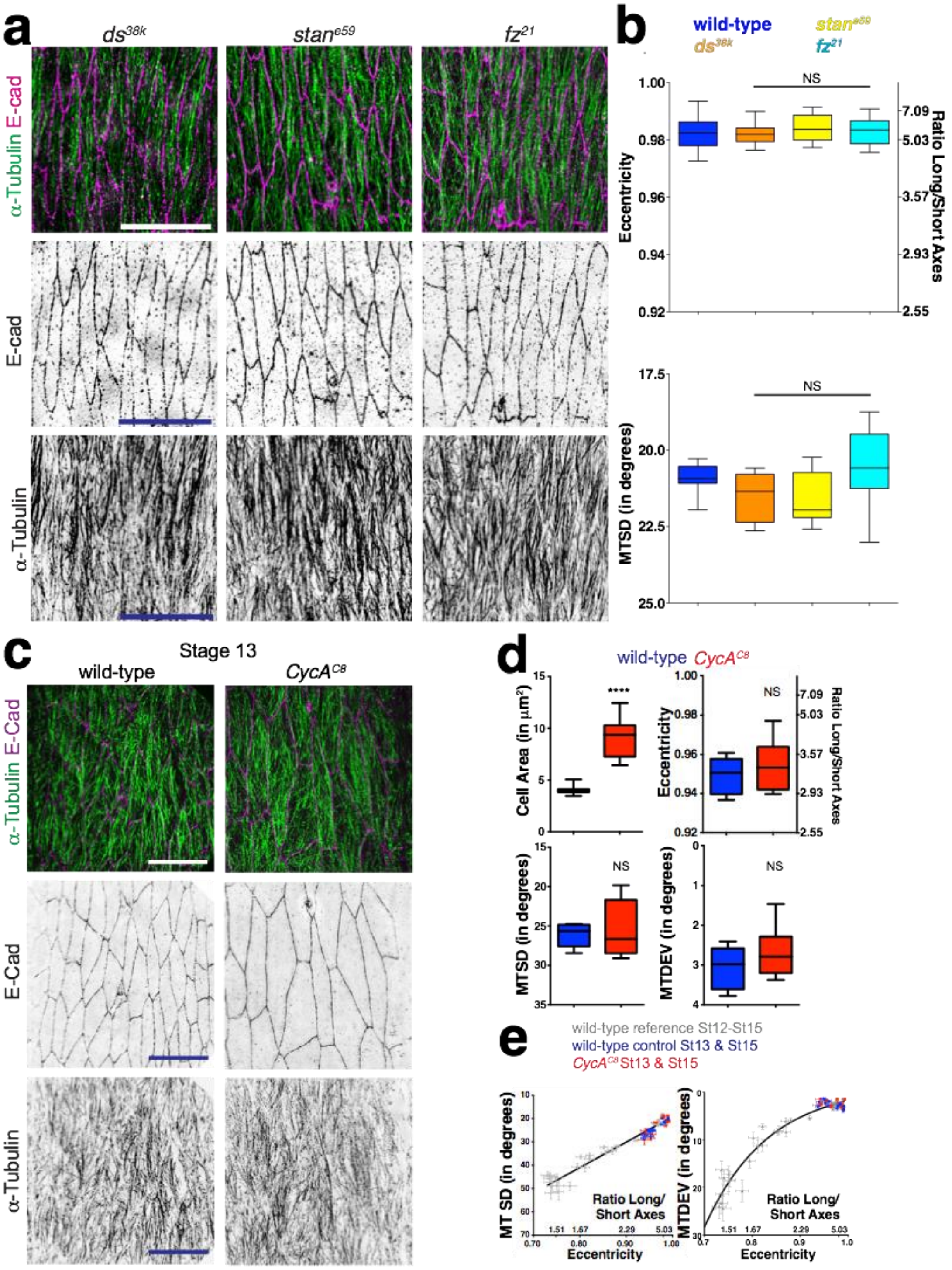

Supplementary Figure 2. (a) Dorso-lateral regions of stage 15 embryonic epidermis, with apical cell outlines revealed with an E-cadherin (E-cad) antibody (magenta) and MTs with an α-Tubulin antibody (green): *dachsous* mutant (*ds*^{38k} ; left), *flamingo/starry night* mutant (*stan*^{e59} ; center) and *frizzled* mutant (*fz*^{21} ; right). Scale

bars - 10 µm. **(b)** Quantification of cell eccentricity and cell's long/short axes ratio (top) and MTSD (bottom). Cells from 12 embryos from 3 (*stan*^{e59}) and 2 (*ds*^{38k},*fz*^{21}) independent experiments were analyzed. Statistical analysis of data combined with data from Figure 1 was performed with one-way ANOVA and Fisher's LSD-post hoc analysis. NS - not significantly different. (**c**) Dorso-lateral regions of stage 13 embryonic epidermis, with apical cell outlines revealed with an E-cadherin (E-cad) antibody (magenta) and MTs with an α-Tubulin antibody (green): wild-type (left) and *CyclinA*^{C8} mutant (*CycA*^{C8}; right). Scale bars - 10 µm. Images in c at lower magnification than a. (**d**) Quantification of cell area, cell eccentricity and cell's long/short axes ratio, MTSD and MTDEV in cells of wild-type (blue) and *CyclinA*^{C8} (red) stage 13 embryos. Cells from 7 embryos per genotype from 3 independent experiments were analyzed. Statistical comparison was performed with unpaired t-test. (**e**) Correlation between either MTSD (top) or MTDEV (bottom), and cell eccentricity is retained when cell area is increased two-fold. Each dot represents the cells from a single embryo (mean ± S.E.M). Cell's long/short axes ratios for eccentricity values of 0.75, 0.8, 0.9 and 0.98 were added in the x axis for reference.

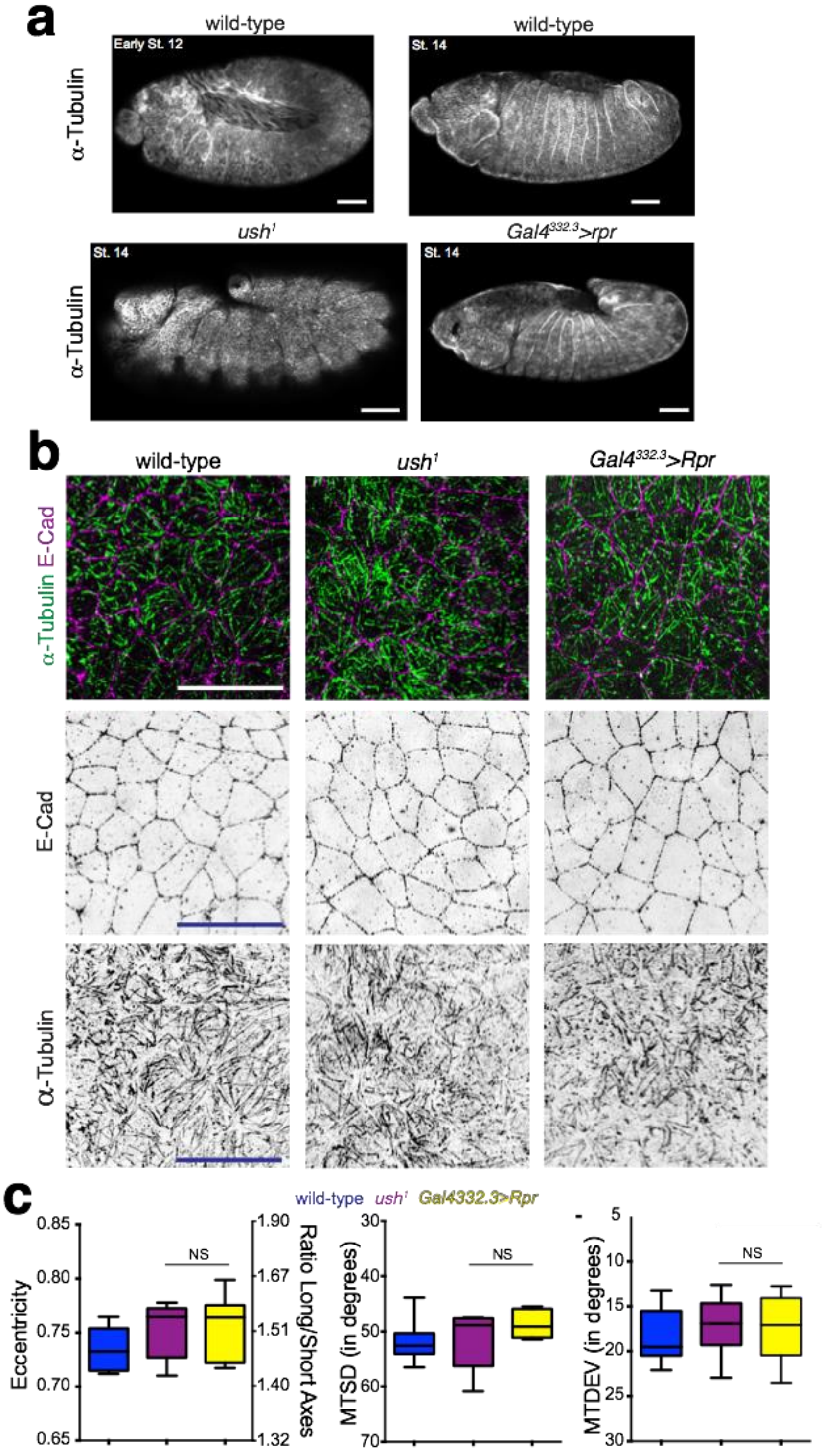

Supplementary Figure 3. (a) Germband retraction defects caused by the *ush*^{1} mutant (lower left) or induction of apoptosis in amnioserosa cells (*Gal4*$^{332.3}$*>rpr*; lower right) in embryos stained for α-Tubulin. Scale bars - 50 μm. **(b)** Representative images of dorsal-lateral epidermis, with apical cell outlines revealed with an E-cadherin (E-cad)

antibody (magenta) and MTs with an α-Tubulin antibody (green), of Early stage 12 wild-type (left), *ush*^{1} mutant (center) and *Gal4*$^{332.3}$*>reaper* (right). Scale bars - 10 μm. **(c)** Quantification of cell eccentricity and cell's long/short axes ratio (left), MTSD (center) and MTDEV (right) shows no defects neither in cell shape nor in microtubule organization prior to the initiation of GBR both in *ush*^{1} and *Gal4*$^{332.3}$*>reaper* embryos. Cells from 7 embryos from 3 independent experiments were analyzed. Statistical analysis was performed with one-way ANOVA and Fisher's LSD post-hoc analysis. NS - not significantly different.

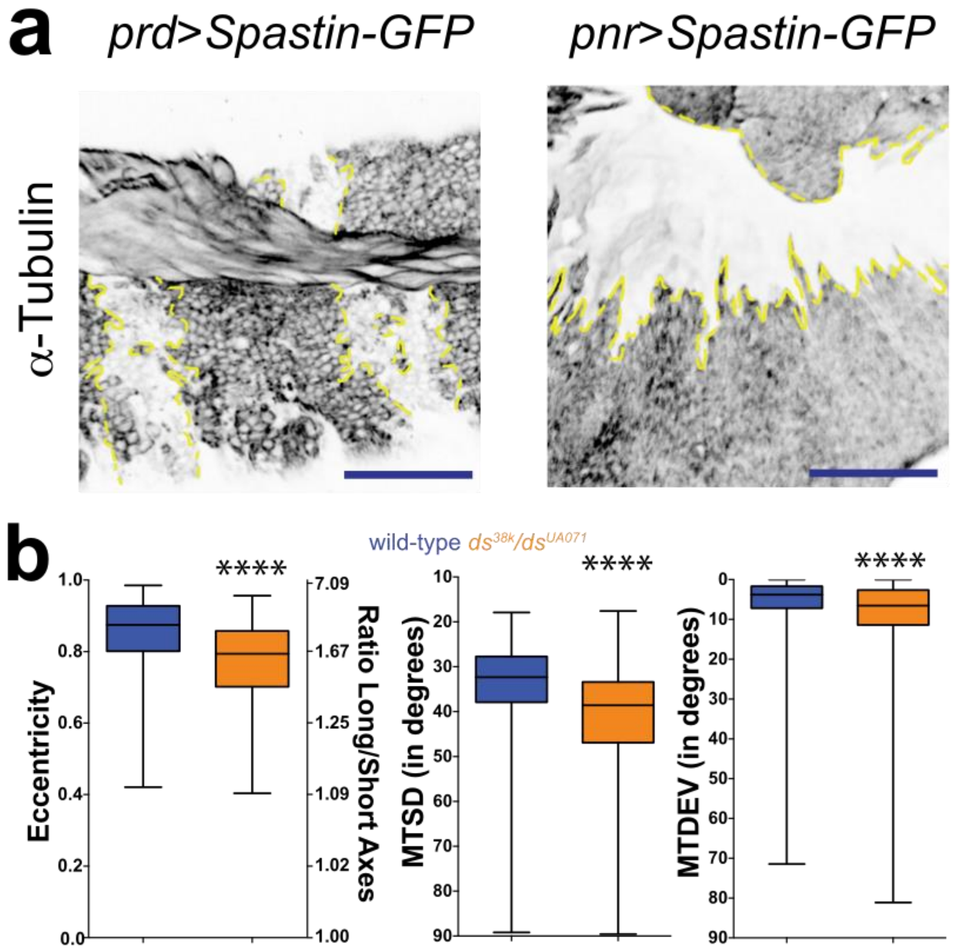

Supplementary Figure 4.

(**a**) Destruction of MTs in early stage 12 embryos by expression of Spastin with either *paired::Gal4* (left) or *pannier::Gal4* (right) stained with anti-α-Tubulin. Yellow dotted line shows boundaries between Spastin expressing and non-expressing cells. Scale bars - 50 μm. (**b**) Quantification of cell eccentricity and cell's long/short axes ratio (left), MTSD (center) and MTDEV (right) shows that both cell elongation and microtubule organization is perturbed in ds^{38k}/ds^{UA071} transheterozygous mutants. 495

cells (wild-type, blue) and 431 cells (*ds*^{38k}/*ds*^{UA071}, orange) from 4 pupae each, obtained in 2 independent experiments, were analyzed.

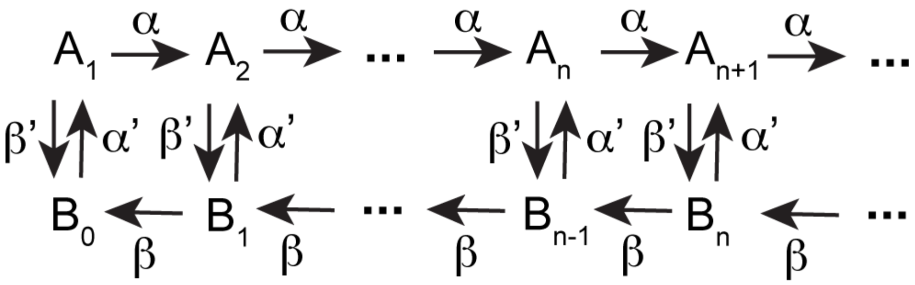

Supplementary Figure 5. Diagram of simulated microtubules states used in the *in silico* model of stochastic microtubule growth.

G_x					G_y				
-2	-1	0	1	2	-2	-3	-4	-3	-2
-3	-2	0	2	3	-1	-2	-3	-2	-1
-4	-3	0	3	4	0	0	0	0	0
-3	-2	0	2	3	1	2	3	2	1
-2	-1	0	1	2	2	3	4	3	2

Supplementary Table 1. 5x5 Sobel operators used to convolve α-Tubulin signal in the Matlab script. Gx and Gy were applied on *x* and *y* coordinates respectively.

Eccentricity	Standard deviation of angle distribution
0.7	40
0.8	30
0.92	22
0.98	22

Supplementary Table 2. Combinations of simulated cell eccentricities with simulated microtubules (100 lines) that were generated at angles randomly sampled from normal distributions with standard deviations of 22º, 30º and 40º.
